# Opinion paper: effectiveness of sirolimus in treating partial Di George syndrome with autoimmune lymphoproliferative syndrome-like features

**DOI:** 10.3389/fped.2023.1242652

**Published:** 2023-11-23

**Authors:** Jutte van der Werff ten Bosch

**Affiliations:** ^1^ Department of Pediatrics, Paola Kinderziekenhuis, Antwerpen, Belgium; ^2^ Department of Medical Hematology, Oncology, HUDERF, Brussels, Belgium

**Keywords:** Di George syndrome, ALPS, treatment, sirolimus, children

## Abstract

Di George syndrome is a disorder well known by pediatricians, caused by deletions in chromosome 22q11.2 and presenting with a wide range of clinical abnormalities, including immunodeficiency. Thymic function is diminished, leading to a decreased output of naive T cells (naïve T-helper cells, naïve T-regulatory cells, naïve cytotoxic T cells) as compared to healthy age matched controls. Immunedysregulation, such as autoimmunity and immunoproliferation are common in these patients, because the lack of a correctly functioning T cell repertoire. treatment of these complication are a challenge in many immunodeficiencies, including Di George syndrome. Gu et al. discuss a case of a patient with 22q11 deletion and lymphoproliferation and treated successfully with Sirolimus. This opinion paper highlights the need to collect information on the treatment of patients with immunoproliferation through sharing of information of individual cases and international cooperation.

## Introduction

Di George syndrome is a disorder well known by pediatricians, caused by deletions in chromosome 22q11.2 and presenting with a wide range of clinical abnormalities, including heart disease, endocrinological abnormalities (hypoparathyroidism), developmental problems (autism, ADHD, characteristic facial abnormalities and cleft lip, musculoskeletal abnormalities etc). Immunodeficiency is a consistent findings in these children, although the severity can vary depending on the remaining thymic tissue. The formation of the thymus is often impaired, leading to thymus hypo- or aplasia, which causes, in turn, a mild or severe T cell penia. Therefore, Di George Syndrome is one of the nearly 500 genetically known inborn errors of immunity (IEI). Thymic function is diminished, leading to a decreased output of naive T cells (naïve T-helper cells, naïve T-regulatory cells, naïve cytotoxic T cells) as compared to healthy age matched controls (1). Receptor repertoires showed skewed V-gene usage for naïve T-helper cells, whereas for naïve cytotoxic T cells, trend towards higher clonality were found.1 With age, T cell counts decrease even further, increasing the possibility of complications (2). This finding can be used to help detect 22q11.2 deletion syndrome patients during neonatal screening for IEI. Interestingly, 22q11 patients also show a perturbation of B-cell subsets, with larger proportions of naïve B cells and lower levels of memory B cells, including switched memory B cells, probably due to the failing T cell help. There may be hypogammaglobulinemia and poor responses to unconjugated Pneumococcal vaccination. For patients with a total absence of the thymus, thymic transplantation is a feasible treatment, but in children with hypoplasia, long term follow up by an immunologist is warranted. Patients are at an increased risk of viral, candida and bacterial infections. As in other patients with diminished immunity, patients with Di George syndrome are also at risk to develop complications. Immunedysregulation, such as autoimmunity and immunoproliferation are common in these patients, because the lack of a correctly functioning T cell repertoire. Autoimmunity is seen in up to 10% of the patients with 22q11.2 (3). Lymphoproliferation has been described in several case reports and can be malignant as well as non-malignant. In review Autoimmune lymphoproliferative syndrome was described more recently. These patients present with chronic benign lymphoproliferation, auto immunity, especially involving the cells of the blood, and an increased risk of lymphoma. The presence of an increased number of a/b double negative T cells in the peripheral blood and lymphoid tissue are pathognomonic for this disease. Besides, hypergammaglobulinemia, increased vitamin B12, increased IL-10 levels and impaired Fas mediated apoptosis can help in the diagnostic process. Mutations in the FAS gene were described as a first cause of this disease, but later a growing list of mutations in other genes were proven to cause ALPS-like disease as well. Nowadays, these entities are referred to as Primary immune regulatory disorders (PIRD). The treatment options for ALPS and PIRD are numerous and depend on the severity of the symptoms. The authors describe a patient with Di George syndrome, lymphoproliferation (chronic adenopathies and splenomegaly) as well as autoimmunity (immune mediated thrombopenia, neutropenia). Further investigations revealed an increased IL-10 level and elevated DN T cells, but no genetic abnormalities associated with chronic lymphoproliferation were found in a whole exome screening. Based on the fact that the presentation of this child mimicked ALPS, they successfully treated the patient with Sirolimus for the cytopenia's and lymphoproliferative symptoms, according to earlier reports (4). Immunedysregulation is a well known complication seen in many types of IEI, and is often a challenge for clinicians treating these patients. Although important progress is being made in the identification of the genes involved in this process, and understanding the exact physiopathology, there are still many questions about the exact mechanisms. Searching for the genetic defect is crucial. Moreover, the “how to treat” question is often a tricky one, as the use of immunomodulatory drugs in patients that are already immunocompromised, can be a challenge. Therefor, it is important to continue to describe and study these patients, preferably using international cohorts, with complete phenotypical and genotypical data. The importance of international collaboration is stressed by the fact that these patients are usually rare. Clinicians should be aware to store material for further research in case biopsies are taken. These cohorts can also benefit the exploration of the different treatment strategies, as a fast growing list of new immunoregulatory drugs, like for example the JAK inhibitors, are added to the already existing, older treatment options, such as corticosteroids and Sirolimus. To investigate what drug to use in a certain individual with immunedysregulation, is one of the many tasks that lay ahead of us. In review References.

## Data Availability

The original contributions presented in the study are included in the article/Supplementary Materials, further inquiries can be directed to the corresponding author.
